# Decluttering Seed Dispersal Modes: Bringing Clarity to Seed Dispersal Ecology

**DOI:** 10.1002/ece3.73203

**Published:** 2026-03-11

**Authors:** Harsh Yadav, Asmita Sengupta, Kim R. McConkey, H. S. Sushma, Shyam S. Phartyal, Takehiro Sasaki

**Affiliations:** ^1^ Graduate School of Environment and Information Sciences Yokohama National University Yokohama Kanagawa Japan; ^2^ SM Sehgal Centre for Biodiversity and Conservation, Ashoka Trust for Research in Ecology and the Environment (ATREE) Bengaluru India; ^3^ BIOTEC, National Science and Technology Development Agency Pathom Thani Thailand; ^4^ Foundation for Ecological Research Advocacy and Learning (FERAL) Morattandi Tamil Nadu India; ^5^ The University of Trans‐Disciplinary Health Sciences and Technology Bengaluru India; ^6^ Department of Forestry Mizoram University Aizawl India; ^7^ Institute for Multidisciplinary Sciences Yokohama National University Yokohama Kanagawa Japan

**Keywords:** classification, polysemy, seed dispersal modes, synonymy, terminological redundancy, terminological uncertainty

## Abstract

Seed dispersal research has expanded significantly over time, leading to a proliferation of terms relating to dispersal modes that has resulted in terminological confusion. This viewpoint identifies the primary concerns in this regard: synonymy (multiple terms used for the same mode) and polysemy (the same term used for distinctly different modes). Such inconsistencies hinder conceptual clarity, impede literature syntheses, and obstruct the practical application of seed dispersal ecology. To address these challenges, we propose two complementary pathways. First, we suggest organizing a world cafe to foster consensus‐building among researchers engaging with seed dispersal ecology. Second, we introduce the Diaspore‐Vector‐Review (DVR) framework as a decision‐support tool to prioritize nomenclature for non‐overlapping dispersal mechanisms rather than agent‐centric definitions of dispersal modes. By refining the branching of subclasses from classical modes into a coherent, hierarchical classification system, we can ensure greater scientific rigor and real‐world impact of seed dispersal research.

## Graphical abstract

Ambiguity and redundancy in seed dispersal modes hinder ecological research and its applications. This viewpoint argues for the need for systematic simplification by proposing a framework for decluttering terminology and preparing a standardized hierarchical classification to resolve inconsistencies and enhance understanding of seed dispersal ecology.

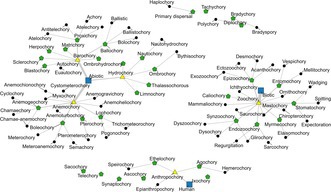

## 1 Background

The documented history of seed dispersal dates back to ~1785 when dispersal categories were first classified (der Van Pijl 1982). However, Ridley's (1930) work stands out as a foundational milestone as he systematically formalized different plant dispersal modes, a framework still widely referenced in contemporary scholarship. Dispersal modes are primarily conceptualized based on diaspore traits and the dispersal vector's interaction with the diaspore (der Van Pijl 1982; Perez‐Harguindeguy et al. 2013). Seed dispersal research has substantially increased in the last four decades (Beckman and Sullivan 2023), with wider utilization of seed dispersal mode terminology (hereafter, dispersal modes). For instance, Sádlo et al. (2018) developed a classification of seed dispersal strategies that used these modes to more accurately define how plants move. Over time, the classical dispersal modes (anemochory, autochory, hydrochory, and zoochory) have branched into diverse subclasses that represent further nuanced mechanisms of seed dispersal (der Van Pijl 1982; Vittoz and Engler 2007).

It is important to expand the terminology space by proposing new concepts when the classical modes fail to adequately capture the nuances of the seed dispersal mechanisms (Green et al. 2022). However, in this viewpoint, we highlight that the introduction, definition, and redefinition of dispersal modes can often lead to terminological ambiguities and redundancies (Green et al. 2022; Soto et al. 2024). Thereafter, we propose ways forward, including a framework for acting as a decision‐support tool for addressing redundancy and ambiguity amongst existing terms as well as coining new ones that can enable meaningful advancements in seed dispersal ecology research and real‐world applications thereof.

## 2 The Seed Dispersal Mode Clutter

**FIGURE 1:**
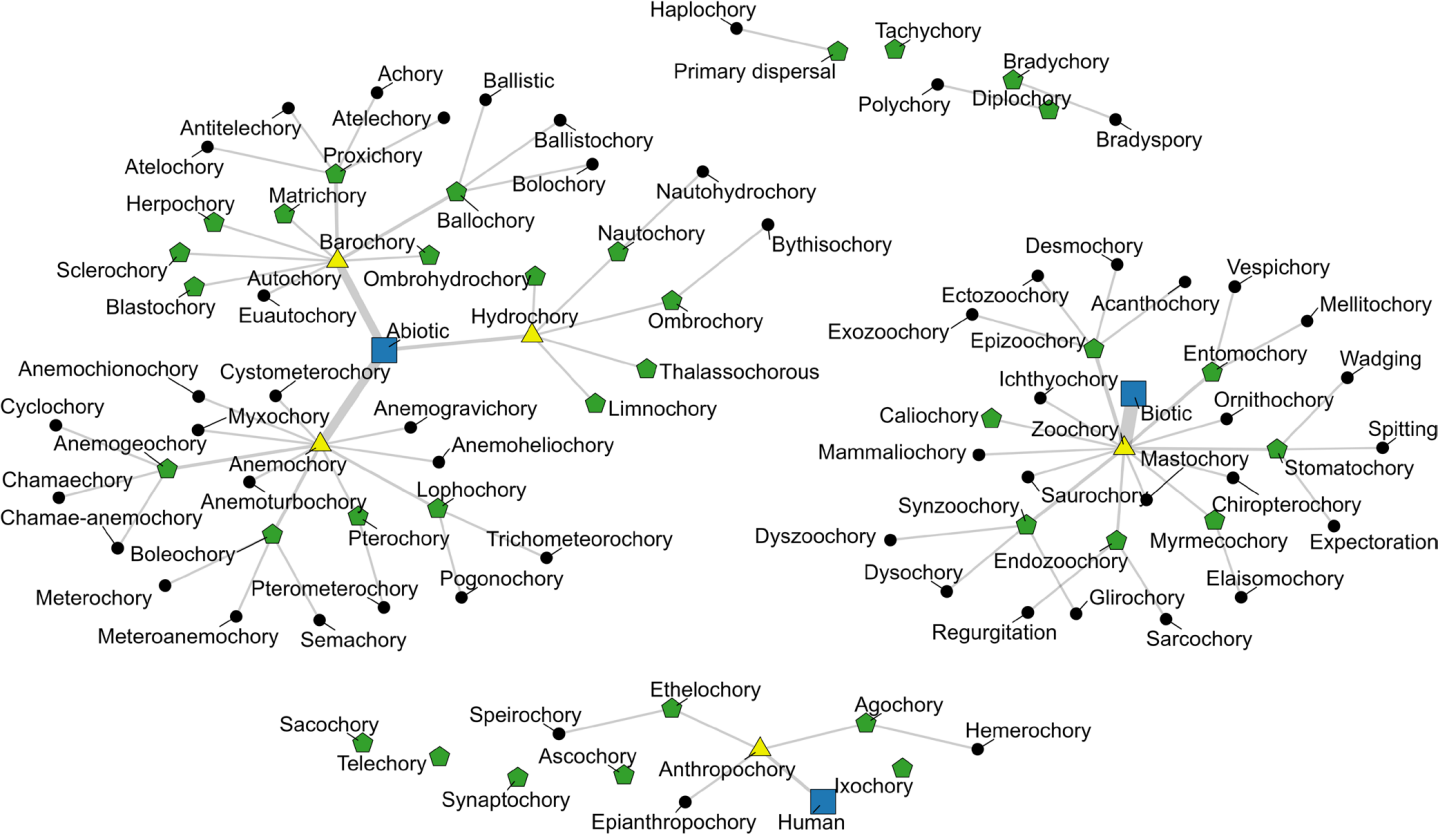
Overlaps in the dispersal modes for the same mechanism. The blue square shows the dispersal category (abiotic, biotic, and human‐mediated). Human‐mediated is not incorporated in the biotic dispersal category as different activities can lead to distinctly different forms of seed dispersal in this category. The yellow triangle shows classical dispersal modes within the dispersal categories. The green pentagons are the subclasses within the classical dispersal modes. The black circles are the dispersal modes overlapping with the subclasses or having similar descriptions as the classical modes. Dispersal modes that are not linked to any of the dispersal categories or classical modes have no clear definition to identify the mechanism or classical mode. Haplochory (primary dispersal) and diplochory (polychory) are not linked to any subclass as they can be used across the dispersal categories.

Drawing from the commonly used modes in seed dispersal research and further complemented by searches on Google Scholar, we first collated an indicative list of dispersal modes (Table S1). The 87 terms compiled hereby included abiotic and biotic as well as human‐mediated dispersal mechanisms. Further, the list provided substantial evidence for the two overarching problems relating to the dispersal mode “clutter”:

- Synonymy: We observed that multiple terms are often used for describing the same dispersal mode (Figure 1), resulting in redundancy. For example, “semachory,” “boleochory,” “meteoranemochory,” and “meteorochory” are all used to describe dispersal by wind; “ballochory,” “ballistochory,” and “bolochory” refer to the explosive propulsion of seeds (Vittoz and Engler 2007; Valdesolo et al. 2022). “Nautochory” and “nautohydrochory” (dispersal by water currents), “pterochory” and “pterometerochory” (diaspores have wings), “dysochory” and “synzoochory” (scatter‐hoarding; der Van Pijl 1982; Sádlo et al. 2018), “elaiosomochory” (Parolly 1998), and “myrmecochory” (dispersal by ants) are some other examples wherein two different terms are used to define the same dispersal mode. Furthermore, “lophochory,” “pogonochory,” and “trichometeorochory” involve the same kinds of diaspores as well as dispersal mode (seeds with hairy structures dispersed by wind; Parolly 1998).
- Polysemy: In contrast, we also found the usage of the same term for distinct dispersal modes. For example, “synzoochory” continues to be used as an umbrella term for seed handling techniques such as scatter‐hoarding, spitting, wadging, or carrying in the mouth. However, Gómez et al. (2019) defined synzoochory as “dispersal of seeds by seed‐caching animals” which makes it equivalent to scatter‐hoarding and distinctly different from “stomatochory” or seed dispersal without swallowing, a mode more suitable for describing other ways of transporting seeds in the mouth (der Van Pijl 1982).

## 3 The Need for Simplification

**FIGURE 2:**
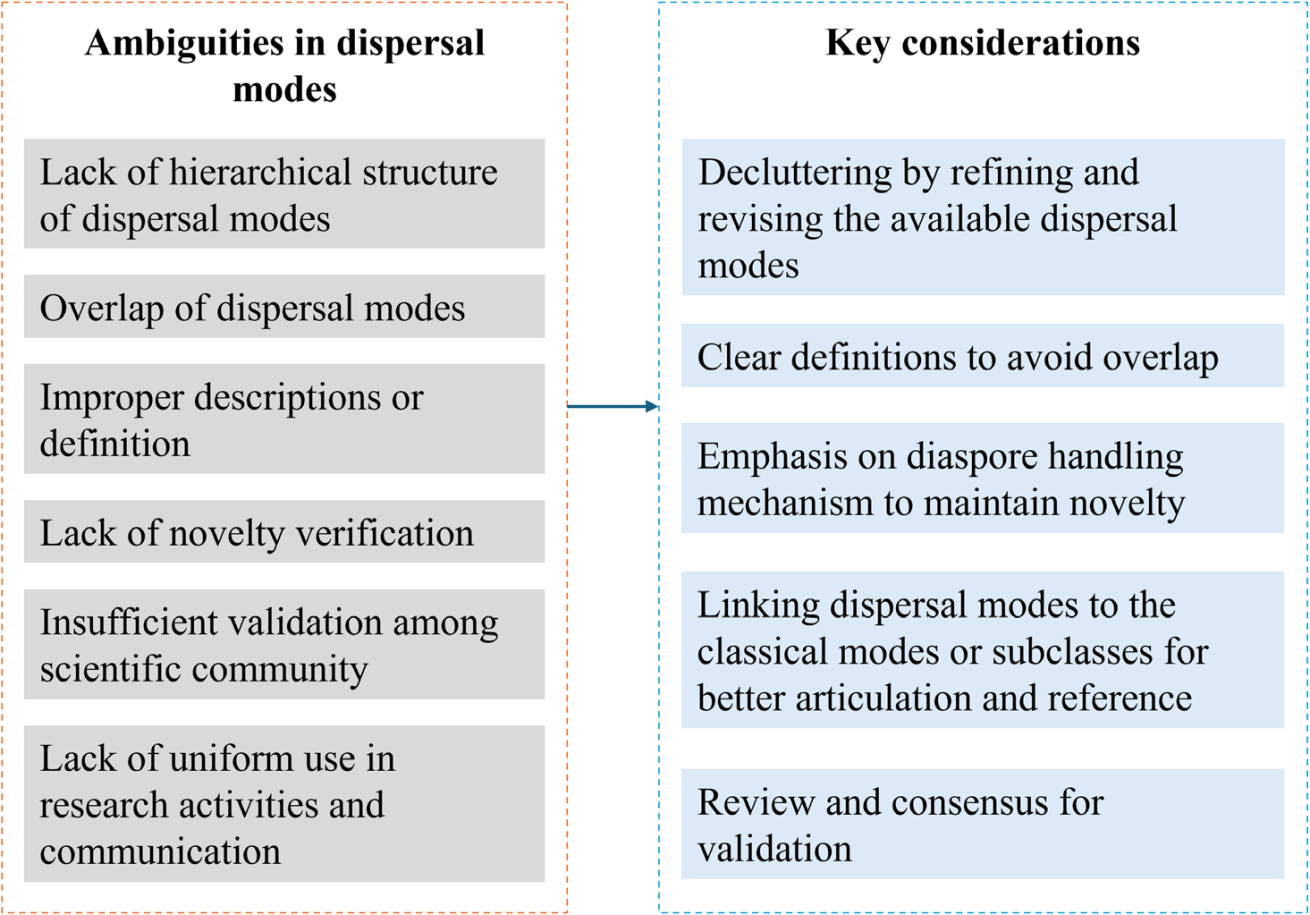
Notable issues with seed dispersal modes and key considerations to resolve and simplify them.

In academia, inconsistency in the usage of terms, manifested in forms of ambiguity and redundancy, can essentially prevent a deeper understanding of concepts (Catford et al. 2009; Soto et al. 2024; Jiménez‐Mejías et al. 2024). Carlo et al. (2025) recently highlighted the ambiguous use of the term “specialization” in plant–animal interactions, drawing attention to the broader challenges posed by inconsistent terminology. Invasion science has similarly recommended simplification of terms to reduce ambiguity (Soto et al. 2024). Inconsistent vegetation terminology has also been shown to complicate studies of European forest biodiversity (Trentanovi et al. 2023). The same problem holds for terminological inconsistencies in seed dispersal ecology (Figure 2). Furthermore, lack of proper definitions and validation from the scientific community further supplements the cluttering in the dispersal mode terminology space.

Additionally, synonymy can make syntheses of findings across studies less efficient; omitting a synonym can result in missing relevant literature, while the proliferation of redundant terms can substantially increase the volume of articles to be screened (Driscoll and Lindenmayer 2012). Polysemy or using the same umbrella term for distinctly different dispersal modes is simply erroneous and creates more confusion, as variations in handling mechanisms have diverse implications for seed viability, transport, deposition, and establishment (Zwolak 2018). Moreover, terminological inconsistencies can hinder real‐world application of seed dispersal research. For example, seed dispersal is directly relevant to practices such as ecological restoration (Gann et al. 2019). Over‐jargonizing dispersal modes can lead to miscommunication of information between researchers, practitioners, and policymakers (Adams et al. 1997).

## 4 Ways Forward

**FIGURE 3:**
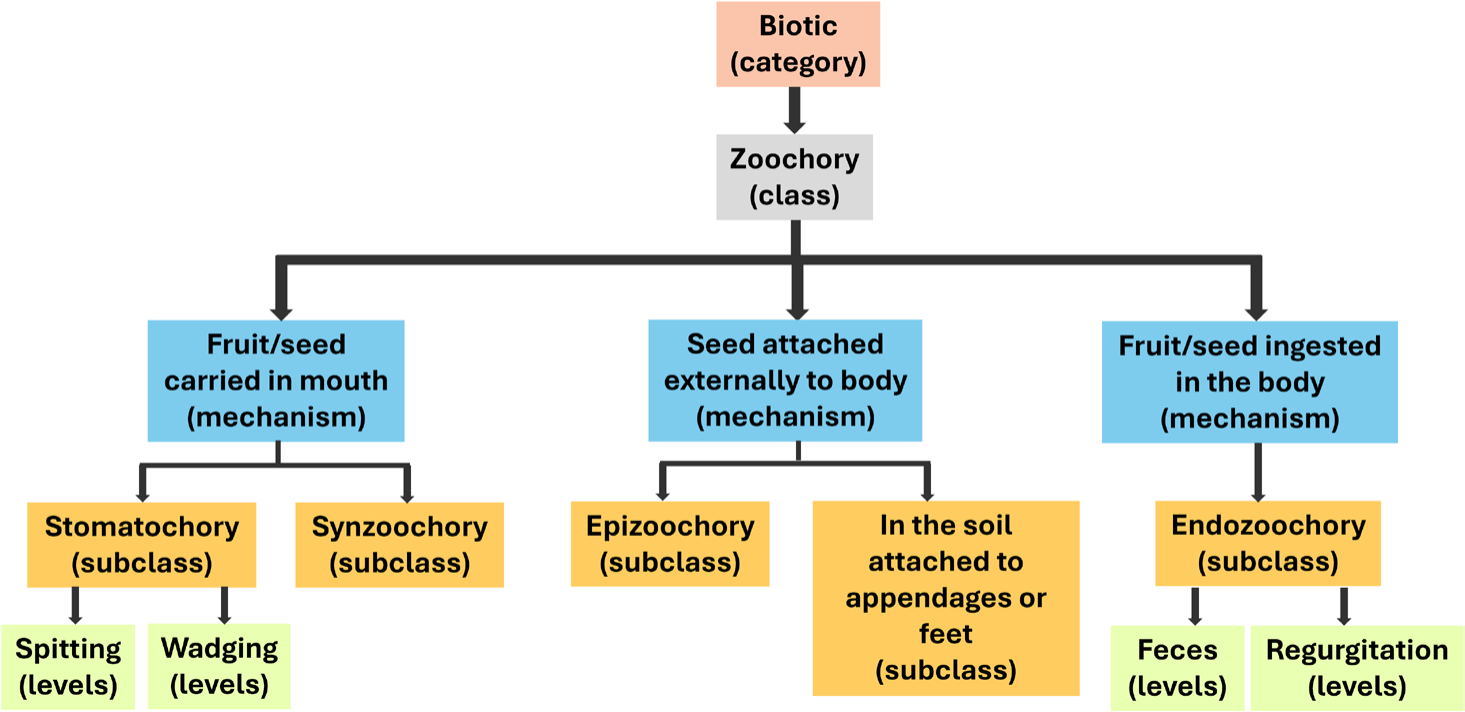
An illustration demonstrating the potential hierarchical classification of seed dispersal modes. This illustration is for representation purposes only. The subclasses may have more levels than shown in this illustration. A separate category of human‐mediated dispersal may account for transportation or other human‐mediated activities leading to dispersal.

Taxonomical standardization of species has made a significant contribution to many academic disciplines which shows that uniformity in accepted nomenclature has wider implications in terms of the advancement of science and communication (Jiménez‐Mejías et al. 2024). To achieve similar standardization for seed dispersal modes, we propose two pathways for clearing the terminology clutter. First, we suggest organizing a World Cafe for researchers engaging with seed dispersal ecology to collectively evaluate the necessity of existing terms and agree on discarding overlapping ones. Similar initiatives, such as that of the Ecological Society of America's Committee on Ecological Nomenclature, have been undertaken in the past to reduce uncertainty in ecological terminology in general (Miyanishi 2014; Herrando‐Pérez et al. 2014). Seed dispersal is studied by researchers from diverse fields using a range of terminologies, and having a common forum for discussion may be particularly useful in this regard. For instance, a specialist in bird behavior and a plant ecologist may realize that they are using different terms for the same process at the World Cafe, allowing them to discuss important nuances relating to their fields and agreeing upon a uniform term. Such discussions will enable a better understanding of dispersal mode nomenclature in different fields, thereby ensuring that the clutter is addressed without losing scientific nuances. The World Cafe may also serve as a platform to develop a hierarchical classification for decluttered seed dispersal modes, drawing from Baskin and Baskin's (2004, 2021) hierarchical seed dormancy classification. The developed classification could comprise the highest level of hierarchy (biotic, abiotic, and human‐mediated) as a dispersal category, with the classical dispersal modes acting as classes. Thereafter, the classification could have novel dispersal mechanism‐based modes as subclasses and levels. For example, under the biotic category, zoochory is the class, stomatochory is the subclass, and spitting and wadging are the levels (Figure 3).

**FIGURE 4:**
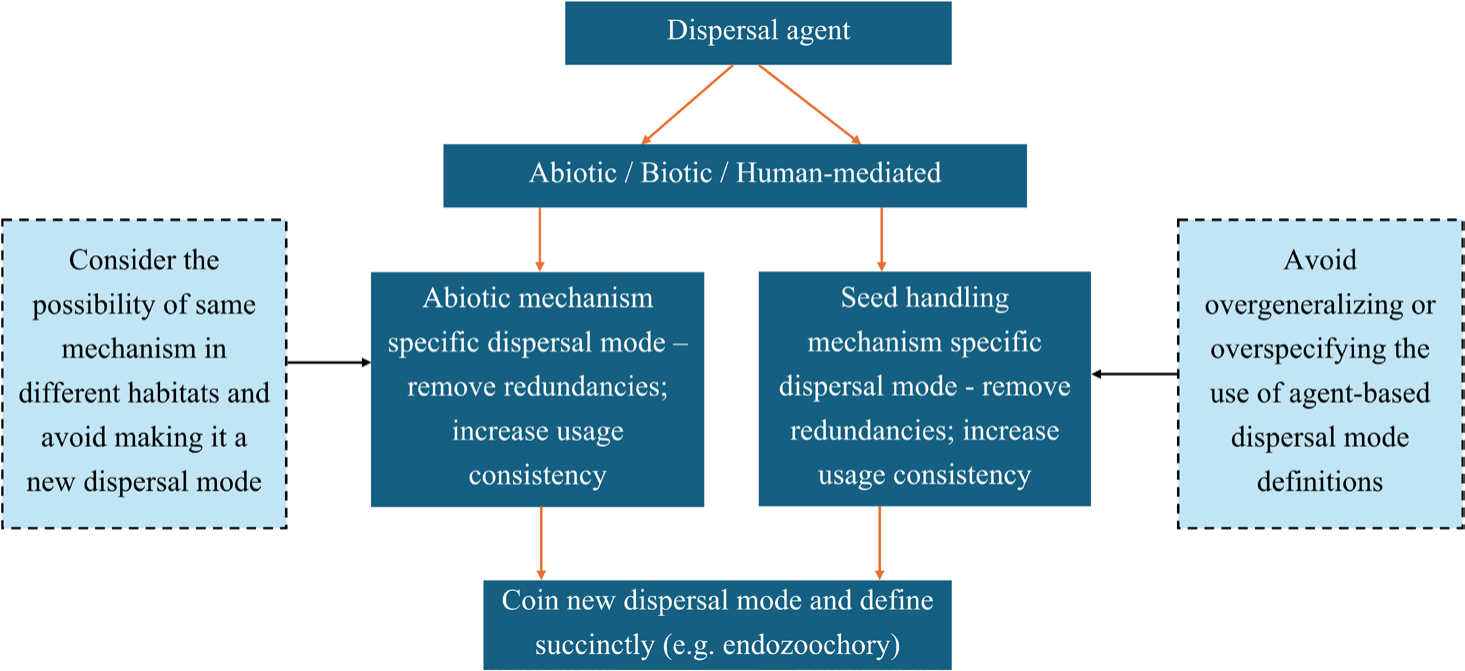
Framework highlighting the removal of redundancy and conceptualizing new dispersal modes.

Second, as a complementary pathway, and more so as a decision‐support tool, we also propose the Diaspore‐Vector‐Review (DVR) framework (Figure 4). This framework is meant to facilitate the removal of redundant terms and assist the conceptualization of new ones only when existing modes fail to capture genuinely novel seed‐handling techniques. It will potentially prevent over‐generalization of agent‐ or habitat‐centric definitions, caution against over‐specificity, and ensure novelty in describing unique seed‐processing interactions. For instance, “caliochory” is specifically defined as seeds carried with nesting materials by birds (Warren et al. 2017). Yet the same dispersal mechanism has been observed in the King Cobra ( *Ophiophagus hannah* ) (Dolia 2020). Moreover, plant materials are used for various purposes other than nest building by animals and carriage of such material can also involve seed dispersal. As per the DVR, synonymy can be prevented by avoiding overspecification with regard to singular vectors or the specific usage of plant material (nest building in this case), thereby providing scope for expanding definitions when required. Similarly, “anemochionochory” is defined as dispersal by horizontal air currents over snowfields (Dixon 1933). Such specificity limits its applicability, since air currents are not unique to a specific habitat and can be both horizontal and vertical; the DVR suggests expanding the definition of the term such that it is neutral to surface or wind direction. Furthermore, this particular dispersal mechanism is actually redundant under DVR, as “anemogravichory” or dispersal by horizontal air currents above the surface of the earth (Dixon 1933) and even the classical mode of “anemochory” (dispersal by wind) can both include “anemochionochory”. In the same way, “hemerochory,” (dispersal of seeds of cultivated plants), “ethelochory” (dispersal via gardening or agriculture), “speirochory” (unintentional introduction of seeds) and “agochory” (car‐mediated seed dispersal) are all different forms of “anthropochory” or human‐mediated seed dispersal. In such scenarios, the DVR suggests that these dispersal modes are better understood based on the handling mechanisms involved (adhesion, ingestion, or transport) that actually drive plant movement rather than having a different term for each human activity.

We acknowledge that at times more informative definitions of existing modes can indeed be useful, such as when clarifying the distinction between secondary dispersal (diaspores moved sequentially by multiple vectors) and diplochory (diaspores dispersed by more than one vector). In such situations, the DVR recommends paying attention to the handling mechanisms involved from seed removal to seedling establishment.

The use of general dispersal mode terms based on broad agent groups such as “anthropochory” or “ornithochory” is justifiable and context dependent. However, caution is also needed in developing overgeneralized terms that can clutter the seed dispersal modes space. We hope that the World Cafe for consensus‐building and the DVR framework for decision support will provide complementary solutions to streamline seed dispersal mode terminology. Thereafter, the hierarchical classification framework for seed dispersal modes will guide the conceptualization and categorization of new terms where necessary. A coherent dispersal mode classification guided by these pathways would strengthen conceptual clarity, enable the standardized integration of seed dispersal modes into trait databases and global research syntheses, and improve communication among researchers, practitioners, and policy stakeholders, thereby improving the practical impact of seed dispersal research.

## Author Contributions

**Harsh Yadav:** conceptualization (equal), data curation (equal), funding acquisition (equal), investigation (equal), methodology (equal), project administration (equal), resources (equal), software (equal), supervision (equal), validation (equal), visualization (equal), writing – original draft (equal), writing – review and editing (equal). **Asmita Sengupta:** conceptualization (equal), data curation (equal), investigation (equal), methodology (equal), supervision (equal), validation (equal), visualization (equal), writing – review and editing (equal). **Kim R. McConkey:** data curation (equal), investigation (equal), methodology (equal), supervision (equal), validation (equal), visualization (supporting), writing – review and editing (equal). **H. S. Sushma:** data curation (equal), investigation (equal), methodology (equal), supervision (equal), validation (equal), visualization (supporting), writing – review and editing (equal). **Shyam S. Phartyal:** data curation (equal), investigation (equal), methodology (supporting), validation (equal), visualization (supporting), writing – review and editing (equal). **Takehiro Sasaki:** funding acquisition (equal), project administration (equal), resources (equal), validation (equal), writing – review and editing (equal).

## Funding

This work was financially supported by a Grant‐in‐Aid for Scientific Research B (Grant no. 25K02041) and the Kajima Foundation (2025‐general research‐19) to T.S.

## Conflicts of Interest

The authors declare no conflicts of interest.

## Supporting information


**Table S1:** The classical and subclasses of seed dispersal modes.

## Data Availability

The curated list of dispersal modes is available as Supporting Information.
