# Supplementary material for: Decluttering Seed Dispersal Modes: Bringing Clarity to Seed Dispersal Ecology
Source: Ecol Evol. 2026 Mar 11;16(3):e73203. doi: 10.1002/ece3.73203 (PMC12978860; doi:10.1002/ece3.73203)
Supplement: Supplementary file 1 — Table S1: The classical and subclasses of seed dispersal modes. [file ECE3-16-e73203-s001.docx]

Table S1: The classical and subclasses of seed dispersal modes. Classical modes represent the four abiotic and biotic natural dispersal modes, with the addition of human-mediated seed dispersal. The subclass dispersal modes branch out from the classical modes. The table also provides a brief description of subclasses. Dispersal modes placed in the ‘others’ at the end of the table either have an unavailable description or are not directly connected to any classical mode.

| **Category** | **Classical mode** | **Dispersal modes** | **Description of dispersal modes** | **Reference** |
| --- | --- | --- | --- | --- |
| **Abiotic** | Anemochory | Anemogeochory | Same as chamae-anemochory | Van der Pijl (1982, Principles of dispersal),  Dixon (1933, *Ecology*) |
|  |  | Chamae-anemochory | The process of dispersal through the tumbling of large parts of plant or whole plant along the surface of the earth | Van der Pijl (1982, Principles of dispersal) |
|  |  | Chamaechory | Wind-based for fruits in grasslands / seeds on snow or dry inflorescence | Vittoz and Engler (2007, *Botanica Helvetica*),  Van der Pijl (1982, Principles of dispersal) |
|  |  | Cyclochory | Distribution of seeds by wind blowing and tumbling the fruiting stages of plants | Parolly (1998, *Phytocoenologia*),  Van der Pijl (1982, Principles of dispersal) |
|  |  | Boleochory | Same as semachory | Vittoz and Engler (2007, *Botanica Helvetica*),  Hitze et al., (2013, *Plant Ecology, Evolution and Systematics*) |
|  |  | Meteoranemochory | Diaspore is blown by wind | Vittoz and Engler (2007, *Botanica Helvetica*),  SER, INSR, RBGK. (2023, *Seed Information Database*) |
|  |  | Meteorochory | Transport of seeds in air | Vittoz and Engler (2007, *Botanica Helvetica*),  Sádlo et al., (2018, *Preslia*) |
|  |  | Semachory | The small seeds without particular features are spread when the fruit is shaken by wind | Vittoz and Engler (2007, *Botanica Helvetica*),  Hitze et al., (2013, *Plant Ecology, Evolution and Systematics*) |
|  |  | Cystometeorochory | Seeds with air-filled structures for wind dispersal. For Orchidaceae, Pyrolaceae, Orobanchaceae | Vittoz and Engler (2007, *Botanica Helvetica*) |
|  |  | Lophochory | Same as pogonochory | Parolly (1998, *Phytocoenologia*) |
|  |  | Pogonochory | Wind dispersal of seeds with special hairy structures | Parolly (1998, *Phytocoenologia*),  Martínez-Orea et al., (2010 *Boletín de la Sociedad Botánica de México*) |
|  |  | Trichometeorochory | Wind dispersal of seeds with special hairy structures. In forest or little efficient plumes | Parolly (1998, *Phytocoenologia*),  LEDA (2024) |
|  |  | Pterochory | Same as pterometerochory |  |
|  |  | Pterometeorochory | Seed dispersal is improved through wings | Martínez-Orea et al., (2010 *Boletín de la Sociedad Botánica de México*),  Vittoz and Engler (2007, *Botanica Helvetica*) |
|  |  | Myxochory | Dispersal with the aid of slime or glue | Parolly (1998, *Phytocoenologia*),  Luftensteiner (1981, Israel Journal of Botany) |
|  |  | Anemogravichory | Distribution by horizontal air currents above the surface of the earth | Dixon (1933, *Ecology*) |
|  |  | Anemochionochory | Distribution by horizontal air currents over the surfaces of snow and ice fields | Dixon (1933, *Ecology*) |
|  |  | Anemoheliochory | Distribution by vertical air currents and later by horizontal currents at higher levels | Dixon (1933, *Ecology*) |
|  |  | Anemoturbochory | Distribution by storms (such as tornadoes) | Dixon (1933, *Ecology*) |
|  |  | Ballochory | Dispersal by explosive mechanism after dehiscence of seed pod | Vittoz and Engler (2007, *Botanica Helvetica*) |
|  | Autochory | Ballistic | Methods originating from parent plant or diaspore; explosive mechanism | Vittoz and Engler (2007, *Botanica Helvetica*),  SER, INSR, RBGK. (2023, *Seed Information Database*) |
|  |  | Ballistochory | Dispersal by mechanical means, where seeds are forcefully ejected from the parent plant. | Vittoz and Engler (2007, *Botanica Helvetica*),  Sendulsky (1993, *Annals of the Missouri Botanical Garden*) |
|  |  | Bolochory | Plants simply drop seeds on the ground or hurl them through explosive mechanisms | Vittoz and Engler (2007, *Botanica Helvetica*),  Valdesolo et al., (2022, *Sustainability*) |
|  |  | Barochory | Dispersal by gravity, where seeds fall directly from the parent plant to the ground or are propelled a short distance upon ripening. | Vittoz and Engler (2007, *Botanica Helvetica*) |
|  |  | Blastochory | Sprawling plant stems carry seed away from parent plant, frequently involving secondary movement by an external vector (Dispersal by vegetative propagules) | Vittoz and Engler (2007, *Botanica Helvetica*) |
|  |  | Herpochory | Self-propelling by structural alterations during successive wet and dry conditions that allow seeds to ‘crawl’ across the substrate (seeds creep on the soil by the movement of organs in a succession of dry and wet conditions) | Vittoz and Engler (2007, *Botanica Helvetica*) |
|  |  | Matrichory | The skeleton of the mother plant with attached seeds facilitates long-distance dispersal | Fllner and Shimda (1981, *Oecologia*) |
|  |  | Proxichory | Seeds and/or diaspores lack morphological characteristics that facilitate long-distance seed dispersal | Fllner and Shimda (1981, *Oecologia*) |
|  |  | Achory | Long-distance seed dispersal hindered by the initial placement of diaspores (at, near, or below the soil surface) or by morphological characteristics of seeds or diaspores | Fllner and Shimda (1981, *Oecologia*),  Van der Pijl (1982, Principles of dispersal) |
|  |  | Antitelechory | Proxichory without antitelechoric characters | Fllner and Shimda (1981, *Oecologia*),  Van der Pijl (1982, Principles of dispersal) |
|  |  | Atelechory | Same as achory | Fllner and Shimda (1981, *Oecologia*) |
|  |  | Atelochory | Same as achory | Fllner and Shimda (1981, *Oecologia*),  Van der Pijl (1982, Principles of dispersal) |
|  |  | Sclerochory | The plant uses a hardened structure or seed coat to propel the seeds | Martínez-Orea et al., (2010 *Boletín de la Sociedad Botánica de México*) |
|  |  | Euautochory | Distribution by self-propelled diaspores such as hygroscopic fruits and seeds | Dixon (1933, *Ecology*) |
|  | Hydrochory | Nautochory | Same as nautohydrochory | Vittoz and Engler (2007, *Botanica Helvetica*),  Van der Pijl (1982, Principles of dispersal) |
|  |  | Nautohydrochory | Dispersal by water currents | Vittoz and Engler (2007, *Botanica Helvetica*),  Van der Pijl (1982, Principles of dispersal) |
|  |  | Ombrochory or Ombrohydrochory | Dispersal by rain splash, where seeds are propelled away from the parent plant by raindrops | Vittoz and Engler (2007, *Botanica Helvetica*) |
|  |  | Bythisochory | Running water may carry different type of seeds with heavy rains | Vittoz and Engler (2007, *Botanica Helvetica*) |
|  |  | Thalassochorous | Saltwater current-assisted dispersal | Vargas et al., (2023, *Biodiversity Data Journal*) |
|  |  | Limnochory | Dispersal in freshwater | Dixon (1933, *Ecology*), Vargas et al., (2023, *Biodiversity Data Journal*) |
| **Biotic** | Zoochory | Dysochory | Same as dyszoochory | Sádlo et al., (2018, *Preslia*) |
|  |  | Dyszoochory | The dispersal of seeds by granivores that accidentally lose them during transport (Scatter hoarding) | Dixon (1933, *Ecology*),  Van der Pijl (1982, Principles of dispersal) |
|  |  | Glirochory | Fruits possess hard skins and lack the smell of typical mammal-dispersed fruits but are still dispersed by rodents. | Van der Pijl (1982, Principles of dispersal), Sernander (1927, *N Acta Reg Soc Sci U ppsaliensis*) |
|  |  | Synzoochory | The deliberate transportation of seeds externally, generally in the mouth, by a granivorous animal, followed by the hoarding of at least a fraction of those seeds | Van der Pijl (1982, Principles of dispersal) |
|  |  | Endozoochory | Dispersal by passing of seeds through gut of an animal and defecating in feces | Vittoz and Engler (2007, *Botanica Helvetica*) |
|  |  | Regurgitation | Seeds are released (vomited out) from the esophagus or stomach without completing the whole digestion process | Vittoz and Engler (2007, *Botanica Helvetica*), Kleyheeg and Leeuwen (2015, *Aquatic Botany*) |
|  |  | Sarcochory | Dispersal by animals (via digestion) of seeds that are contained in fleshy fruits | Vittoz and Engler (2007, *Botanica Helvetica*), LEDA (2024), Martínez-Orea et al., (2010, *Boletín de la Sociedad Botánica de México*) |
|  |  | Epizoochory | Dispersal by exterior attachment to the animal's body | Van der Pijl (1982, Principles of dispersal) |
|  |  | Acanthochory | Seed dispersal by animals, mainly in the fur | Van der Pijl (1982, Principles of dispersal),  Martínez-Orea et al., (2012, *Revista mexicana de biodiversidad*) |
|  |  | Desmochory | Seed can be spiny or having adhesive organs that allow them to stick on a transport agent | Van der Pijl (1982, Principles of dispersal),  Tchiengué (2013) |
|  |  | Ectozoochory | Same as epizoochory | Van der Pijl (1982, Principles of dispersal),  Coughlan et al., (2019, *Frontiers in Ecology and Evolution*) |
|  |  | Exozoochory | Same as epizoochory | Van der Pijl (1982, Principles of dispersal),  Coughlan et al., (2019, *Frontiers in Ecology and Evolution*) |
|  |  | Stomatochory | Transport in an animal’s mouth without swallowing the seed | Van der Pijl (1982, Principles of dispersal)  Sádlo et al., (2018, *Preslia*) |
|  |  | Spitting | Similar to expectoration | Corlett and Lucas (1990, *Oecologia*) |
|  |  | Expectoration | Seed dispersal by spitting | Burtt and Salisbury (1929, *Journal of Ecology*) |
|  |  | Wadging | Intact discarding of seeds after maneuvering the fruit in the lower lip and extracting the juices | Lambert (1999, *American Journal of Physical Anthropology*) |
|  |  | Mammaliochory | Seed dispersal by mammals | Chang et al., (2014, *The Scientific World Journal*) |
|  |  | Mastochory | Mammals other than bats | Hilje et al., (2015, *Tropical Conservation Science*) |
|  |  | Chiropterochory | Dispersal by bats | Vittoz and Engler (2007, *Botanica Helvetica*),  Van der Pijl (1982, Principles of dispersal) |
|  |  | Ornithochory | Dispersal specifically by birds | Vittoz and Engler (2007, *Botanica Helvetica*),  Van der Pijl (1982, Principles of dispersal) |
|  |  | Ichthyochory | Dispersal by fish | Vittoz and Engler (2007, *Botanica Helvetica*),  Van der Pijl (1982, Principles of dispersal) |
|  |  | Saurochory | Seed dispersal by reptiles | Platt et al., (2013, *Journal of Zoology*) |
|  |  | Entomochory | Seed dispersal by insects | Li Vigni and Melati (1999, *Acta botanica gallica*) |
|  |  | Mellitochory | Dispersal by bees (fruits of some species like Eucalyptus that produce resin as an attractant for bees | Li Vigni and Melati (1999, *Acta botanica gallica*),  Poschold et al., (2025) |
|  |  | Vespichory | Dispersal of seed by wasps | Li Vigni and Melati (1999, *Acta botanica gallica*),  Jules (1996, *American Midland Naturalist*) |
|  |  | Myrmecochorous/Myrmecochory | Same as elaiosomochory | Vittoz and Engler (2007, *Botanica Helvetica*) |
|  |  | Elaisomochory | Dispersal by ants | Vittoz and Engler (2007, *Botanica Helvetica*),  Parolly (1998, *Phytocoenologia*) |
|  |  | Caliochory | Seeds transported attached to the plant material used for making nests | Warren et al., (2017, *Plant Ecology*) |
| **Human** | Anthropochory | Agochory | Seeds travelling hidden in goods cars, soil under soles, with hay | Vittoz and Engler (2007, *Botanica Helvetica*) |
|  |  | Hemerochory | The spreading of diaspores by the indirect activity of man | Vittoz and Engler (2007, *Botanica Helvetica*),  Van der Pijl (1982, Principles of dispersal),  Hejný and Jehlík (1972, *Folia Geobotanica & Phytotaxonomica*) |
|  |  | Ethelochory | Plants or seeds sold for agriculture and gardening mostly in urban or cultivated areas | Vittoz and Engler (2007, *Botanica Helvetica*) |
|  |  | Speirochory | Plants or seeds used mostly in urban or cultivated areas | Van der Pijl (1982, Principles of dispersal),  Vittoz and Engler (2007, *Botanica Helvetica*) |
|  |  | Epianthropochory | Transport of diaspores upon the body of humans | Van der Pijl (1982, Principles of dispersal),  Dixon (1933, *Ecology*) |
| **Others** |  | Ascochory | No description found | Martínez-Orea et al., (2012, *Revista mexicana de biodiversidad*) |
|  |  | Bradychory | Same as bradyspory | Thanos (2004) |
|  |  | Bradyspory | The phenomenon of delayed dispersal | Fllner and Shimda (1981, *Oecologia*) |
|  |  | Diplochory | Same as polychory | Van der Pijl (1982, Principles of dispersal) |
|  |  | Polychory | Scope of dispersal by multiple mechanisms or vectors | Van der Pijl (1982, Principles of dispersal) |
|  |  | Primary dispersal | Same as haplochory | Vander Wall et al., (2005, *Ecology*) |
|  |  | Haplochory | Dispersal mediated by a single standard dispersal vector | Vander Wall et al., (2005, *Ecology*),  Nathan et al., (2008, *Trends in Ecology and Evolution*) |
|  |  | Ixochory | No description found | Martínez-Orea et al., (2012, *Revista mexicana de biodiversidad*) |
|  |  | Sacochory | No description found | Dansereau and Lems (1957),  Martínez-Orea et al., (2012, *Revista mexicana de biodiversidad*) |
|  |  | Secondary dispersal | A multistep process with two or more phases, which involve different dispersers that usually extend the distance from the seed’s parent plant (e.g. anemohydrochory) | Padilla et al., (2012, *Journal of Ecology*) |
|  |  | Synaptochory | The diaspore has morphological characteristics facilitating long-distance seed dispersal | Fllner and Shimda (1981, *Oecologia*) |
|  |  | Telechory | Seeds or diaspores possess morphological characters facilitating long-distance dispersal of seeds (e.g., barbs, pappi, fleshy fruits) | Fllner and Shimda (1981, *Oecologia*) |
|  |  | Tachychory | Distribution of diaspores immediately after ripening | Dixon (1933, *Ecology*) |

**References used for collating seed dispersal modes**

- Burtt, B. D., & Salisbury, E. J. (1929). A Record of Fruits and Seeds Dispersed by Mammals and Birds from the Singida District of Tanganyika Territory. Journal of Ecology, 17(2), 351–355. <https://doi.org/10.2307/2256047>
- Chang, L., He, Y., Yang, T., Du, J., Niu, H., & Pu, T. (2014). Analysis of herbaceous plant succession and dispersal mechanisms in deglaciated terrain on Mt. Yulong, China. The Scientific World Journal, 2014(1), 154539. <https://doi.org/10.1155/2014/154539>
- Corlett, R.T., & Lucas, P.W. (1990). Alternative seed-handling strategies in primates: seed-spitting by long-tailed macaques (Macaca fascicularis). Oecologia 82, 166–171. <https://doi.org/10.1007/BF00323531>
- Coughlan, N. E., Dickey, J. W., Cuthbert, R. N., Kelly, T. C., Jansen, M. A., & Dick, J. T. (2019). Driver's seat: Understanding divergent zoochorous dispersal of propagules. Frontiers in Ecology and Evolution, 7, 70. <https://doi.org/10.3389/fevo.2019.00070>
- Dansereau, P., & Lems, K. (1957). The grading of dispersal types in plant communities and their ecological significance.
- Dixon, H. (1933). The technique of plant distribution. Ecology, 14, 226-228
- Fllner, S., & Shmida, A. (1981). Why are adaptations for long-range seed dispersal rare in desert plants?. Oecologia, 51, 133-144.
- Hilje, B., Calvo-Alvarado, J., Jiménez-Rodríguez, C., & Sánchez-Azofeifa, A. (2015). Tree species composition, breeding systems, and pollination and dispersal syndromes in three forest successional stages in a tropical dry forest in Mesoamerica. Tropical Conservation Science, 8(1), 76-94.
- Hintze, C., Heydel, F., Hoppe, C., Cunze, S., König, A., & Tackenberg, O. (2013). D3: the dispersal and diaspore database–baseline data and statistics on seed dispersal. Perspectives in Plant Ecology, Evolution and Systematics, 15(3), 180-192. <https://doi.org/10.1016/j.ppees.2013.02.001>
- Jules, E. S. (1996). Yellow jackets (Vespula vulgaris) as a second seed disperser for the myrmecochorous plant, Trillium ovatum. American Midland Naturalist, 367-369.
- Kleyheeg, E., & van Leeuwen, C. H. (2015). Regurgitation by waterfowl: an overlooked mechanism for long-distance dispersal of wetland plant seeds. Aquatic Botany, 127, 1-5. <https://doi.org/10.1016/j.aquabot.2015.06.009>
- Lambert, J. E. (1999). Seed handling in chimpanzees (Pan troglodytes) and redtail monkeys (Cercopithecus ascanius): Implications for understanding hominoid and cercopithecine fruit‐processing strategies and seed dispersal. American Journal of Physical Anthropology: The Official Publication of the American Association of Physical Anthropologists, 109(3), 365-386.
- LEDA. (2024). Traitbase Appendix E: Glossary of terms. Available at <https://uol.de/f/5/inst/biologie/ag/landeco/download/LEDA/Standards/Leda-App-E.pdf>
- Li Vigni, I., & Melati, M. R. (1999). Examples of seed dispersal by entomochory. Acta botanica gallica, 146(2), 145-156.
- Luftensteiner, H. W. (1981). Myxochory in four different plant communities in Austria. Israel Journal of Botany, 30(2), 95-98. <https://doi.org/10.1080/0021213X.1981.10676911>.
- Martínez-Orea, Y., Castillo-Argüero, S., Guadarrama-Chávez, M. P., & Sánchez, I. (2010). Post-fire seed bank in a xerophytic shrubland. Boletín de la Sociedad Botánica de México, (86), 11-21. <https://www.scielo.org.mx/scielo.php?script=sci_arttext&pid=S0366-21282010000100002>
- Martínez-Orea, Y., Castillo-Argüero, S., Hernández-Apolinar, M., Guadarrama-Chávez, M. P., & Orozco-Segovia, A. (2012). Seed rain after a fire in a xerophytic shrubland. Revista mexicana de biodiversidad, 83(2), 447-457.
- Nathan, R., Schurr, F. M., Spiegel, O., Steinitz, O., Trakhtenbrot, A., & Tsoar, A. (2008). Mechanisms of long-distance seed dispersal. Trends in ecology & evolution, 23(11), 638-647. <https://doi.org/10.1016/j.tree.2008.08.003>
- Padilla, D. P., González‐Castro, A., & Nogales, M. (2012). Significance and extent of secondary seed dispersal by predatory birds on oceanic islands: the case of the Canary archipelago. Journal of Ecology, 100(2), 416-427. <https://doi.org/10.1111/j.1365-2745.2011.01924.x>
- Parolly, G. (1998). Phytosociological studies on high mountain plant communities of the South Anatolian Taurus mountains 1. Scree plant communities (Heldreichietea): a synopsis. Phytocoenologia, 233-284.
- Platt, S. G., Elsey, R. M., Liu, H., Rainwater, T. R., Nifong, J. C., Rosenblatt, A. E., ... & Mazzotti, F. J. (2013). Frugivory and seed dispersal by crocodilians: an overlooked form of saurochory?. Journal of Zoology, 291(2), 87-99. <https://doi.org/10.1111/jzo.12052>
- Poschlod, P., Mašková, T., Chen, S. C., Phartyal, S., Rosbakh, S., et al. (2025). A handbook for standardised measurements of regenerative plant functional traits.
- Sádlo, J., Chytrý, M., Pergl, J., & Pyšek, P. (2018). Plant dispersal strategies: A new classification based on the multiple dispersal modes of individual species. <https://doi.org/10.23855/preslia.2018.001>.
- Sendulsky, T. (1993). First report of ballistochory in the Poaceae. Annals of the Missouri Botanical Garden, 518-521. <https://doi.org/10.2307/2399798>
- SER, INSR, RBGK. (2023). Seed Information Database (SID). Available at <https://ser-sid.org/>
- Sernander R (1927) Zur Morphologie und Biologie der Diasporen. N Acta Reg Soc Sci U ppsaliensis, U ppsala
- Slavomil Hejný, & Vladimír Jehlík. (1972). Hemerochorous Dispersal of Adventitious Plants from the Viewpoint of Frequency of Different Ways of Introduction: A Proposal of Terminology. Folia Geobotanica & Phytotaxonomica, 7(1), 91–93
- Tchiengué, B. (2013). Secondary successions after shifting cultivation in a dense tropical forest of southern Cameroon (Central Africa) (Doctoral dissertation, Frankfurt am Main, Johann Wolfgang Goethe-Univ., Diss., 2012).
- Thanos, C. A. (2004). Bradychory—The coining of a new term. In Proceedings 10th medecos conference, Millpress, Rotterdam.
- Thomson, F. J., Moles, A. T., Auld, T. D., Ramp, D., Ren, S., & Kingsford, R. T. (2010). Chasing the unknown: predicting seed dispersal mechanisms from plant traits. Journal of Ecology, 98(6), 1310-1318. <https://doi.org/10.1111/j.1365-2745.2010.01724.x>
- Valdesolo, T., Del Vecchio, S., & Buffa, G. (2022). Patterns of seed dispersal in coastal dune plant communities. Sustainability, 14(17), 10983.
- Van der Pijl, L. (1982). Principles of dispersal. Berlin: SpringerVerlag.
- Vander Wall, S. B., Kuhn, K. M., & Beck, M. J. (2005). Seed removal, seed predation, and secondary dispersal. Ecology, 86(3), 801-806. <https://doi.org/10.1890/04-0847>
- Vargas, P., Heleno, R., & Costa, J. M. (2023). EuDiS-A comprehensive database of the seed dispersal syndromes of the European flora. Biodiversity Data Journal, 11, e104079. <https://doi.org/10.3897/BDJ.11.e104079>
- Vittoz, P., & Engler, R. (2007). Seed dispersal distances: a typology based on dispersal modes and plant traits. Botanica Helvetica, 117, 109-124. <https://doi.org/10.1007/s00035-007-0797-8>
- Warren, R. J., Love, J. P., & Bradford, M. A. (2017). Nest-mediated seed dispersal. Plant Ecology, 218, 1213-1220. <https://doi.org/10.1007/s11258-017-0763-5>.
